# What mechanism of niche segregation allows the coexistence of sympatric sibling rhinolophid bats?

**DOI:** 10.1186/1742-9994-9-30

**Published:** 2012-11-13

**Authors:** Egoitz Salsamendi, Inazio Garin, Inmaculada Arostegui, Urtzi Goiti, Joxerra Aihartza

**Affiliations:** 1 Department of Zoology and Animal Cell Biology, Faculty of Science and Technology, University of the Basque Country UPV/EHU, Sarriena z/g, Leioa E-48940, The Basque Country; 2 Department of Applied Mathematics, Statistics and Operational Research, Faculty of Science and Technology, University of the Basque Country UPV/EHU, Sarriena z/g, Leioa E-48940, The Basque Country

**Keywords:** Chiroptera, Coexistence, Diet, Foraging habitat, Morphology, Sibling species, *Rhinolophus*

## Abstract

**Introduction:**

Our purpose was to assess how pairs of sibling horseshoe bats coexists when their morphology and echolocation are almost identical. We collected data on echolocation, wing morphology, diet, and habitat use of sympatric *Rhinolophus mehelyi* and *R*. *euryale*. We compared our results with literature data collected in allopatry with similar protocols and at the same time of the year (breeding season).

**Results:**

Echolocation frequencies recorded in sympatry for *R*. *mehelyi* (mean = 106.8 kHz) and *R*. *euryale* (105.1 kHz) were similar to those reported in allopatry (*R*. *mehelyi* 105–111 kHz; *R*. *euryale* 101–109 kHz). Wing parameters were larger in *R*. *mehelyi* than *R*. *euryale* for both sympatric and allopatric conditions. Moths constitute the bulk of the diet of both species in sympatry and allopatry, with minor variation in the amounts of other prey. There were no inter-specific differences in the use of foraging habitats in allopatry in terms of structural complexity, however we found inter-specific differences between sympatric populations: *R*. *mehelyi* foraged in less complex habitats. The subtle inter-specific differences in echolocation frequency seems to be unlikely to facilitate dietary niche partitioning; overall divergences observed in diet may be explained as a consequence of differential prey availability among foraging habitats. Inter-specific differences in the use of foraging habitats in sympatry seems to be the main dimension for niche partitioning between *R*. *mehelyi* and *R*. *euryale*, probably due to letter differences in wing morphology.

**Conclusions:**

Coexistence between sympatric sibling horseshoe bats is likely allowed by a displacement in spatial niche dimension, presumably due to the wing morphology of each species, and shifts the niche domains that minimise competition. Effective measures for conservation of sibling/similar horseshoe bats should guarantee structural diversity of foraging habitats.

## Introduction

According to the ecomorphological paradigm, species with similar morphology should exhibit similarities in behaviour and ecology [1]. This prediction, however, raises the possibility of competition between such species when they occur in sympatry. Inter-specific competition takes place when two (or more) species with similar ecological requirements consume resources that are limited in supply [2]. Nevertheless, the stable coexistence of competitors will be possible if their respective niches differ sufficiently [3].

Niche differentiation is easy to conceptualise as a consequence of inter-specific competition. However, concrete evidence in support of it is difficult to acquire, because the demonstration of niche differentiation *per se* does not necessarily indicate anything about the contribution of competition. Removal or demographic response experiments, if adequately designed, may demonstrate a cause-effect relationship between niche differentiation and inter-specific competition [4,5]. However, these experiments are inappropriate for rare, elusive, *K*-selected, or endangered species, and currently, non-disruptive, more inductive approaches are the only practical alternatives. These alternative approaches usually compare morphology, behaviour, and ecology of two (or more) species which occur under allopatric and sympatric conditions and assume that any niche displacement is directly associated with competition [6].

Morphologically similar species are numerous among bats [7], and the number is increasing as molecular tools uncover cryptic species-complexes comprising genetically isolated taxa [8,9]. An excellent illustration is the discovery that the most abundant and best-known European bat species, the pipistrelle, is in fact a cryptic complex of two species: the common pipistrelle (*Pipistrellus pipistrellus*) and the soprano pipistrelle (*P*. *pygmaeus*) [10,11]. Despite being morphologically almost indistinguishable, ecological data show a marked divergence in ecological requirements [12,13], contradicting ecomorphological predictions.

Bats depend on flight as their principal means of locomotion, so wing morphology greatly influences foraging behaviour [14]. Additionally, most bats use echolocation to obtain information about their environment, the precision of which depends on the structure of the echolocation signal [15]. Thus, characterisation of bats’ wing morphology and echolocation signals, facilitate inferences about ecological significance. For example, bats with narrow, pointed wings usually emit low-frequency calls and tend to forage in open spaces, whereas bats with broad, rounded wings usually emit high-frequency signals and tend to forage in cluttered space (*i*.*e*. forests) [16,17]. Some species in the families Vespertilionidae and Molossidae, exhibit flexibility in the structure of search-phase echolocation signals [18,19], enabling them to access a wide variety of habitats [20]. Other species, for example in the families Mormoopidae and Rhinolophidae, emit unique, stereotyped signal structures with little quantitative variation; these bats are more rigid in the use of foraging habitats [21,22].

Rhinolophids have a highly specialised auditory system that discriminates the modulated echoes of the beating wings of insects (*e*.*g*. moths) from unmodulated background echoes [23]. Based on their auditory system and wing morphology, horseshoe bats are classified as narrow-space, flutter-detecting foragers: they are specialised to catch slow-flying insects very close to or within vegetation [15]. Horseshoe bats consist of a single genus, *Rhinolophus*, with 77 recognised species distributed exclusively in the Old World [7]. Although the group is diverse, its morphological uniformity is striking compared to other families. This uniformity is mirrored in the numerous, morphologically similar, and frequently confused pairs of sympatric species [24-26]. Segregation in habitat use and diet have been proposed as major mechanisms for niche differentiation in sympatric bat species [27-30]. However, it remains unclear what promotes niche differentiation in sympatric horseshoe bats, as there are few ecological or behavioural studies (but see [22]), and none of potential sibling species (*sensu*[31]).

The Mehely’s (*Rhinolophus mehelyi* Matschie, 1901) and the Mediterranean (*R*. *euryale* Blasius, 1853) horseshoe bats can be considered as sibling species (*i*.*e*. they are morphologically similar and share a recent common ancestor [32,33]). *R*. *mehelyi* and *R*. *euryale* are two cave-dwelling species whose distributions overlap extensively in the Mediterranean basin [25]. Radio-tracking studies show that in allopatric conditions both forage in and along forest edges [34,35]. A preliminary study suggests that in sympatric conditions they tend to segregate foraging habitats with *R*. *euryale* found in more dense woodlands [36]. However, the small sample size and the coarse resolution of locations attained by triangulation of that study [36], as well as the lack of information on diet, limit any firm conclusion about niche differentiation. *R*. *mehelyi* and *R*. *euryale* emit similar echolocation signal structures differing only in the frequency of maximum energy, 107 and 104 kHz, respectively [37,38]. Although broad differences in frequency may facilitate dietary niche differentiation, frequency differences between both species may be too small to allow any dietary differentiation [22]. So far no diet data have been collected for sympatric populations, but moths (Lepidoptera) are both species’ main prey in allopatry [34,39]. The question remains, how do sibling horseshoe bat species coexist when echolocation signal structure is unlikely to result in differences in diet and their morphology is almost identical?

To address this question we investigated echolocation, wing morphology, diet, and habitat use of *R*. *mehelyi* and *R*. *euryale* in sympatric conditions during breeding season in the Iberian Peninsula, and compared our results with previous literature in allopatric populations. To make results comparable we focused on data gathered in the Iberian Peninsula during breeding season. Our aim was to ascertain the mechanism(s) of coexistence. We predicted that dietary niche differentiation is not likely to occur but rather that coexistence stems from spatial niche differentiation, with slight differences in wing parameters as the prominent contributing factor. Lower wing loading and aspect ratio values in *R*. *euryale* should make it better adapted to forage in more cluttered space, facilitating habitat partitioning.

## Results and discussion

### Echolocation and wing parameters

We measured echolocation frequencies and wing parameters from 16 *R*. *mehelyi* and 24 *R*. *euryale* individuals in sympatry. We found significant differences in resting frequency (RF) and wing parameters between *R*. *mehelyi* and *R*. *euryale* (multivariate ANOVA: *F* = 15.9; *d*.*f*. = 7, 30; *p* < 0.001). The interaction between species and sex was not significant (multivariate ANOVA: *F* = 2.0; *d*.*f*. = 7, 30; *p* = 0.110). *R*. *mehelyi* emitted significantly higher RF than *R*. *euryale*, 106.8 ± 1.5 kHz (mean ± SD) versus 105.1 ± 1.2 kHz, respectively (Table 1). Mass, forearm length, wingspan, wing area, aspect ratio, and wing loading were greater for *R*. *mehelyi* (Table 1). The echolocation frequencies and wing parameters recorded in this study (sympatric condition) are in accordance to previous published data on allopatric conditions [17,24,25,38,40-42].

**Table 1:** **Echolocation and wing parameters in *R***. ***mehelyi *and *R***. ***euryale *in sympatry**

	***R.mehelyi *(16)**	***R.euryale *(24)**	***F***	***p***
RF (kHz)	106.8 ± 1.5	105.1 ± 1.2	37. 2	***
Body mass (g)	15.3 ± 1.5	12.0 ± 1.7	51.1	***
Forearm length (mm)	50.7 ± 1.1	47.9 ± 0.8	36.6	***
Wingspan (mm)	32.0 ± 1.3	30.0 ± 0.9	20.2	***
Wing area (mm^2^)	164.5 ± 9.7	150.3 ± 9.4	13.2	*
Aspect ratio	6.2 ± 0.5	6.0 ± 0.4	6.5	*
Wing loading (N/m^2^)	9.1 ± 1.5	7.9 ± 0.9	15.2	*

Mean values ± standard deviations for resting frequency (RF), body mass, forearm length, wingspan, wing area, aspect ratio, and wing loading of *R*. *mehelyi* and *R*. *euryale* in sympatric condition. Sample sizes are shown in parentheses. *F* values for variable effects between species and significances are provided (*** = *p* < 0.001; * = *p* < 0.05; ns = not significant).

### Diet and prey size

In sympatry, we analyzed 221 faecal pellets collected from 16 *R*. *mehelyi* and 31 *R*. *euryale* individuals (mean = 4.7 pellets/bat). We identified eight prey categories belonging to five insect orders. The bulk of the diet of *R*. *mehelyi* and *R*. *euryale* were composed by moths (Lepidoptera), which represented 95% and 85% of average prey volume respectively. Neuroptera (Families Chrysopidae, Myrmeleontidae and Hemerobidae) were important prey for *R*. *euryale*, representing more than 12% of the average volume (Table 2). *R*. *mehelyi* showed a less diverse diet, consuming three prey categories, whereas *R*. *euryale* consumed up to seven categories (Table 2). There were slight but statistically significant differences between species (two-way ANOVA: *F* = 1.72; *d*.*f*. = 7; *p* = 0.04); *R*. *mehelyi* consumed significantly more moths and fewer green lacewings (Chrysopidae) than *R*. *euryale* (two-way ANOVA: *F* = 5.32; *d*.*f*. = 1; *p* = 0.03 and *F* = 15.70; *d*.*f*. = 1; *p* < 0.001, respectively). We measured 35 moth tarsus fragments from the faeces of 30 *R*. *mehelyi* individuals, and 24 fragments from 22 *R*. *euryale* individuals. Mean estimated body length of moths did not differ between the two species: *R*. *mehelyi* = 14.1 ± 2.1 mm; *R*. *euryale* = 12.7 ± 2.1 mm (Mann-Whitney: *U* = 294.5; *d*.*f*. = 57; *p* = 0.06).

**Table 2:** **Diet of *R***. ***mehelyi *and *R***. ***euryale *in sympatry vs allopatry**

**Prey category**	**Sympatry**		**Allopatry**	
	***R. mehelyi *(16)**	***R. euryale *(31)**	***R. mehelyi *(43)**^**1**^	***R. euryale *(43)**^**2**^
Lepidoptera	94.9	86.1	98.3	92.7
Chrysopidae		4.9	0.6	2.5
Myrmeleontidae		5.1		2.4
Hemerobiidae		2.3		
Brachycera		0.3	0.2	0.7
Tipulidae			0.5	0.5
Scarabeidae		1.2		
Chrysomelidae	4.9			
Hemiptera	0.2	0.1		
Psocoptera				1.1
Unidentified			0.4	0.3

Mean percentage volume (%) of the prey categories found in faeces of *R*. *mehelyi* and *R*. *euryale* in sympatric and allopatric conditions. Sample sizes are shown in parentheses. ^1^ Source: Salsamendi *et al*. [39]; ^2^ Source: Goiti *et al.*[34].

In allopatry, Salsamendi *et al*. analysed 215 faecal pellets from 43 *R*. *mehelyi* individuals (mean = 5 pellets/bat) [39], and Goiti *et al*. analized 237 pellets from 43 *R*. *euryale* individuals (5.5 pellets/bat) [34]. The diets of *R*. *mehelyi* and *R*. *euryale* were also composed by moths, which represented 98% of average prey volume for *R*. *mehelyi*[39] and 93% of average prey volume for *R*. *euryale*[34]. Neuroptera (Families Chrysopidae and Myrmeleontidae) again showed to be important for *R*. *euryale*, representing more than 5% of the average volume (Table 2). *R*. *mehelyi* showed again a less diverse diet, consuming 4 prey categories [39], whereas *R*. *euryale* consumed 6 prey categories [34]. There were significant inter-specific differences in diet (two-way ANOVA: *F* = 2.37; *d*.*f*. = 6; *p* = 0.02); *R*. *mehelyi* consumed more moths and fewer green lacewings (Chrysopidae) than *R*. *euryale* (two-way ANOVA: *F* =9.68; *d*.*f*. = 1; *p* < 0.01 and *F* = 5.29; *d*.*f*. = 1; *p* = 0.03, respectively). We found no information on the prey sizes consumed in allopatric conditions.

*R*. *mehelyi* displaying higher echolocation frequencies, consumed more moths than *R*. *euryale* both in sympatric and allopatryc conditions, in accordance with the allotonic frequency hypothesis, which predicts that bats displaying higher-frequency echolocation signals should consume a higher proportion of moths [43]. However, we argue that differences in echolocation frequencies used by *R*. *mehelyi* and *R*. *euryale* are presumably too small to produce dietary resource partitioning and might not be responsible for the diet divergence [22,37,38]. Instead, we propose that observed differences in diet may stem from differences in availability of insect prey in the foraging habitats that bats use (please refer to habitat use subsection below) [44]. For instance, the diet and prey selection in *R*. *euryale* varies with seasonal prey availability [34]. Differences in diet of *R*. *mehelyi* and *R*. *euryale* observed elsewhere can also be explained by local variability of available prey [34,37,44-47].

The average moth size of *R*. *mehelyi* was slightly larger than that for *R*. *euryale* (difference = 1.4 mm). This may be attributable to bat’s body size thresholds. Although prey size differences were not statistically significant, the larger overall size of *R*. *mehelyi* suggests that it may select larger prey as a response to differences in skull and jaw biometrics [22]. We also propose that differences in the bats’ energy budget relative to prey detection, handling, and consumption may be an alternative explanation. The slightly larger body size of *R*. *mehelyi* would reduce manoeuvrability, making larger prey worth hunting.

### Habitat use

**Figure 1:**
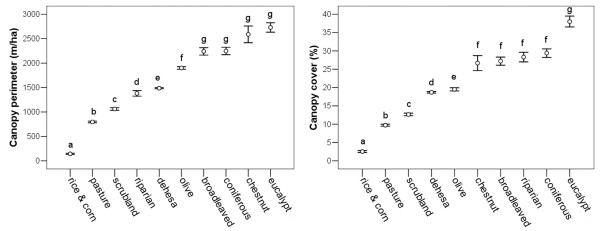
**Structural complexity of habitat types in sympatry.** Mean values and 95% confidence intervals for canopy perimeter (left) and canopy cover (right) among habitat types in sympatric conditions. Habitat types are ranked from lowest to highest values of canopy perimeter and cover as surrogates for structural complexity. Different letters denote significant differences (Dunnett T3 post-hoc tests) between habitat types.

For the four environmental variables measured in sympatry we found strong associations between habitat type and canopy perimeter (ANOVA: *F* = 1513.9; *d*.*f*. = 1, 9; *p* < 0.001) and between habitat type and canopy cover (ANOVA: *F* = 1083.3; *d*.*f*. = 1, 9; *p* < 0.001). We also found a strong correlation between canopy perimeter and canopy cover (Pearson’ s *r*_xy_ = 0.789; *p* < 0.001). Rice and corn fields, pastures, and scrublands were the habitat types with the lowest canopy perimeter and cover values, whereas eucalypt plantations had the highest values (Figure 1).

**Figure 2:**
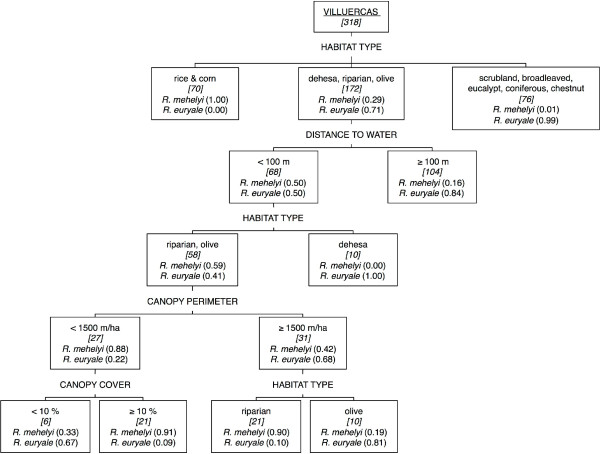
**CART model for habitat use by *R***. ***mehelyi *and *R***. ***euryale *in sympatry.** Classification and regression tree model for differential habitat use by *R*. *mehelyi* and *R*. *euryale* in sympatric conditions in Villuercas (Spain). The response variables are presence of *R*. *mehelyi* and *R*. *euryale* and the explanatory variables are habitat type, distance to water, canopy perimeter, and canopy cover. Top node represents training data set (60% of entire data set), non-terminal nodes represent data splits, and terminal nodes represent homogeneous classes. All nodes are labelled with their determining variable’s value/category and the number of foraging fixes for both species in each group (italicised and in brackets), as well as probability of finding a foraging *R*. *mehelyi* or *R*. *euryale* (in parentheses). An illustration of how to use the CART model: in any site where habitat type is dehesa, olive grove, or riparian forest (follow middle branch in the 1^st^ node *habitat type*), if distance to water is more than 100 m (follow right branch in the 2^nd^ node *distance to water*), the resulting probability of this site being used for foraging by *R*. *mehelyi* is 0.16, whereas the probability for *R*. *euryale* is 0.84.

In sympatry, 12 *R*. *mehelyi* individuals and 13 *R*. *euryale* individuals provided data to feed the CART analysis (Additional file 1: Appendix A). Overall, we obtained 542 foraging fixes, 214 for *R*. *mehelyi* and 328 for *R*. *euryale*. The CART model assigned habitat type as the first splitting variable to distinguish foraging site preferences between *R*. *mehelyi* and *R*. *euryale*, followed by distance to water, then canopy perimeter, and finally canopy cover (Figure 2). Rice and corn fields were exclusively used by *R*. *mehelyi* (foraging probability = 1.00), whereas *R*. *euryale* avoided this habitat type (foraging probability = 0.00). Scrublands, broadleaved woodlands, eucalypt plantations, coniferous plantations, and chestnut groves were used almost exclusively by *R*. *euryale* (foraging probability = 0.99), whereas both species used dehesas, riparian forests, and olive groves. Neither species used pastures. Generally, foraging probability for both species increased as distance to water decreased; distance to water bodies from foraging sites did not differ between species (ANOVA: *F* = 0.14; *d*.*f*. = 1,1; *p* = 0.71). The CART model showed an overall high predictive power, correctly classifying 88.3% of the validation fixes (*c* = 0.86; confidence interval = 0.81–0.91); specifically, it correctly classified 72.8% of *R*. *mehelyi* validation fixes and 99.2% for *R*. *euryale*.

In allopatry, Salsamendi *et al*. radio-tracked 25 *R*. *mehelyi* individuals [35] and Goiti *et al*. radio-tracked 15 *R*. *euryale* individuals [34]. Overall, 398 foraging fixes were obtained for *R*. *mehelyi*, whereas 373 were obtained for *R*. *euryale*[34,35]. *R*. *mehelyi* showed preference for eucalypt plantations, riparian forests and broadleaved woodlands [35], whereas *R*. *euryale* showed preference for hedgerows, broadleaved woodlands and isolated trees [34]. None of the species used pastures for foraging.

**Figure 3:**
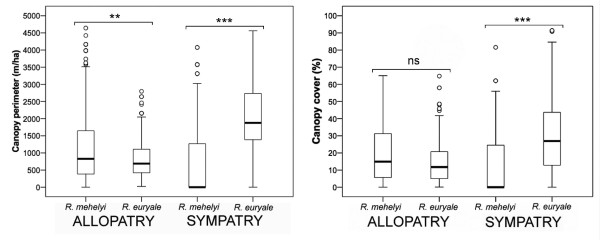
**Niche shift between *R*. *mehelyi *and *R*. *euryale *in allopatry vs sympatry.** Distribution box-plots for canopy perimeter (left) and canopy cover (right) values used by *R*. *mehelyi* and *R*. *euryale* in allopatric and sympatric conditions; significance is also provided (*** = *p*<0.001; ** = p<0.01; ns = not significant). Bold lines indicate median and boxes encompass interquartile range (IQR, 25%–75%). T-bars encompass values within 1.5 IQR from median and the circles represent outliers.

In allopatric conditions, we found no differences in the use of canopy cover values between *R*. *mehelyi* and *R*. *euryale* (Tukey post-hoc comparison: *p* = 0.113; Figure 3), but there were differences in the use of canopy perimeter values between *R*. *mehelyi* and *R*. *euryale* (Tukey post-hoc comparison: *p* = 0.004). However, in sympatric conditions we found significant inter-specific differences in both canopy cover and canopy perimeter values (Tukey post-hoc comparisons: *p* < 0.001 and *p* < 0.001, respectively; Figure 3).

Our results show that, in sympatry, both species exhibit similar but not completely identical foraging habitat preferences. We found that exclusive foraging habitats for *R*. *euryale* included broadleaved woodlands, scrublands, eucalypt and coniferous plantations, and chestnut groves, where *R*. *mehelyi* was rarely encountered; in contrast, exclusive foraging habitats for *R*. *mehelyi* included rice and corn fields. In a semiarid ecosystem as in the Mediterranean, where water availability is limited, habitats associated with water likely support high insect abundances [48,49]. Crops requiring irrigation or flooding, such as rice and corn fields, may offer alternative foraging sites when other habitats dry up during summer [35]. We note that while both species foraged close to water bodies, where moths are readily found, foraging sites diverged in structural complexity.

### Foraging behaviour

Stereotyped foraging behaviour of both species was characterised by continuous back-and-forth flights close to vegetation; *R*. *mehelyi* flew at 0.5–1.5 m from to tree canopies, linear structures, and isolated trees, whereas *R*. *euryale* flew within 0.5 m, repeatedly plunging in and even diving through the branches. We also observed *R*. *mehelyi* flying continuously back-and-forth in open environments, close (30-50 cm) to ground vegetation, whereas *R*. *euryale* was never recorded in open environments.

Mean straight-line distance from the roost to foraging sites was significantly larger in *R*. *mehelyi* than in *R*. *euryale*; 19.2 ± 7.0 km and 4.8 ± 2.4 km, respectively (Mann-Whitney: *U* = 12.0; *d*.*f*. = 23; *p* < 0.001). Maximum individual flight distance for *R*. *mehelyi* was 29.1 km and 10.1 km for *R*. *euryale*. There was no inter-specific difference in foraging home range (*R*. *mehelyi* = 242 ± 341 ha vs. *R*. *euryale* = 153 ± 316 ha; Man-Whitney: *U* = 53.5; *d*.*f*. = 22; *p* = 0.29). Mean and maximum commuting distances for *R*. *euryale* are similar to previously published data [36,50-53]. However, mean and maximum distances to foraging sites were nearly five-fold greater for *R*. *mehelyi*[35,36]. This contrasts with previous studies of *R*. *mehelyi* and other congeners in which commuting distances were short, consistent with the slow, butterfly-like flight of rhinolophids [17,35,36,54]. *R*. *mehelyi* is theoretically better-adapted to commute longer distances than *R*. *euryale*, as its wing parameters should make it a more efficient flyer for long distances [17,55]. Indeed, Rainho and Palmeirim [56] reported commuting distances for *R*. *mehelyi* similar to those we found (up to 22 km), confirming that these bats are capable of flying long distances to foraging sites. The commuting distances we recorded are likely a consequence of landscape configuration, where favoured foraging areas are particularly distant from the roost.

### Niche breadth and overlap

Habitat breadth did not differ between *R*. *mehelyi* (B = 2.81 ± 0.56) and *R*. *euryale* (B = 4.1 ± 0.77; *t* = -0.63; *p* = 0.55, randomisation test after 10000 iterations). However, the magnitude of intra-specific habitat overlap was significantly larger in *R*. *mehelyi* (FT = 0.47 ± 0.38) than in *R*. *euryale* (FT = 0.33 ± 0.35; *t* = -2.14; *p* = 0.01). The magnitude of intra-specific habitat overlap for each species was significantly larger than the inter-specific overlap (FT = 0.21 ± 0.27; *t* = 2.66; *p* = 0.009, and *t* = 5.64; *p* < 0.001, respectively). Trophic niche breadth was larger for *R*. *mehelyi* than *R*. *euryale*, B = 1.34 ± 0.59 and B = 1.12 ± 0.13, respectively (*t* = -2.2; *p* = 0.03). Magnitude of intra-specific trophic overlap was lower in *R*. *euryale* than in *R*. *mehelyi*, FT = 0.88 ± 1.4 versus FT = 0.93 ± 0.19, respectively (*t* = 3.39; *p* < 0.001). Inter-specific trophic overlap, FT = 0.89 ± 0.17, was significantly lower than the intra-specific overlap of *R*. *mehelyi* (*t* = 2.3; *p* = 0.03) but significantly higher than that of *R*. *euryale* (*t* = -0.9; *p* = 0.02).

### Niche segregation

Although differences exist in the diet of *R*. *mehelyi* and *R*. *eurale*, the bulk of the diet of both species in sympatric and allopatric conditions consist of moths [34,39,46,47]. Moreover, in sympatry we found that intra-specific trophic overlap of *R*. *euryale* was lower than inter-specific trophic overlap, making the species’ coexistence through a dietary niche dimension unstable [57]. Although molecular identification of moths eaten is required prior to discarding any functional difference of diet patterns e.g. [58], segregation in foraging habitats appears to be a more likely scenario for niche segregation of these bat species in sympatry. Subtle differences in echolocation frequencies between both species seem to be unlikely to facilitate dietary partitioning. Divergences observed in diet may be explained as a consequence of differential prey availability among foraging habitats.

As narrow-space, flutter-detecting foragers, *R*. *mehelyi* and *R*. *euryale* are both adapted to forage in structurally complex, highly cluttered environments [15]. Based on the species’ similarities in wing morphology and echolocation parameters, their respective niches should largely overlap in relation to habitat use. In allopatric populations of southern Iberian Peninsula, *R*. *mehelyi* forages not only in dehesas but also in riparian forests, broadleaved woodlands, and eucalypt plantations [35]. In areas with an Atlantic climate in the northern Iberian Peninsula, *R*. *euryale* forages not only in habitats with high structural complexity but also semi-open habitats, predominantly hedgerows, woodland edges and isolated trees in meadows [34]. However, foraging habitats reported here (*i*.*e*. under sympatric conditions) for *R*. *mehelyi*, seem to be more restricted to open spaces, whereas *R*. *euryale* uses more cluttered ones. This is best illustrated in Figure 3, where there is a significant niche displacement in the use of canopy cover and canopy perimeter values from allopatric to sympatric conditions. All of this suggests that the potential niches related to habitat use are likely broader than those we observed; *i*.*e*. under sympatric conditions, the two species occupy narrower habitat niches, most likely a consequence of inter-specific interactions that lead to a niche displacement. This niche displacement is conditioned by subtle differences in wing morphology and shifts the niche domains that may limit competition between both species.

## Conclusions

This study shows that *R*. *mehelyi* and *R*. *euryale* exhibit similar ecologies in sympatry yet showing subtle but significant differences. The most pronounced ecological difference is their divergent use of foraging habitats that contrasts with the low divergence in diet. This is reflected by the low magnitude of inter-specific habitat overlap compared to inter-specific trophic overlap.

Differences in echolocation call frequencies between *R*. *mehelyi* and *R*. *euryale* are presumably too small to allow any dietary partitioning and, accordingly, there were no inter-specific differences in the size of moth consumed. Diet divergences between *R*. *mehelyi* and *R*. *euryale* in both sympatric and allopatric conditions may stem from differences in prey availability among habitats used for foraging. As a general pattern, there were virtually no differences in the use of foraging habitats between *R*. *mehelyi* and *R*. *euryale* in allopatric conditions. However, under sympatric conditions, both species display a shift in the use of foraging habitats: *R*. *euryale* forages in more complex environments characterized by higher values of canopy cover and canopy perimeter, whereas *R*. *mehelyi* forages in less cluttered/more open environments.

Differences in the use of foraging habitats, rather than differences in diet, seem to be the main mechanisms for resource partitioning between *R*. *mehelyi* and *R*. *euryale*, as a consequence of slight differences in wing morphology. Coexistence between sympatric sibling horseshoe bats is likely allowed by a displacement in spatial niche dimension, presumably due to morphological features of each species, and shifts the niche domain that minimise competition. Effective measures for conservation of sibling/similar horseshoe bats should guarantee structural diversity of foraging habitats so coexisting species have enough “space” to segregate their respective niches.

## Materials and methods

### Literature review

To make results comparable between sympatric (this study) and allopatric conditions, literature review for allopatric data followed the next criterions: 1) study period should be the same time of the year (*i*.*e*. breeding season); 2) study areas should be geographically as close as possible (preferably the Iberian Peninsula); 3) protocols used to measure echolocation, wing parameters, and diet should be similar and results comparable; and 4) radio-tracking protocols must be identical (*e*.*g*. homing-in) and sampling effort comparable. Following these criterions, our literature review outputted seven publications for echolocation and wing parameters [17,24,25,38,40-42], whereas for diet and habitat use we only found three publications [34,35,39]. The following subsections describe the protocols used for data and sample collection in sympatric conditions. Protocols used to data and sample collection in allopatry will be added when needed.

### Study area and bat capture

Fieldwork was conducted during breeding season (June–July) in 2007 at Sierra de Las Villuercas mountain range, in Extremadura, Spain (UTM 30S 2924 4359), south-western Iberian Peninsula. In the area, maternity colonies of *R*. *mehelyi* and *R*. *euryale* roost together in three adjacent, disused train tunnels. Emergence video-counts revealed that in 2007 at least 190 *R*. *mehelyi*/*R*. *euryale* individuals roosted in the three tunnels altogether; the small inter-specific differences in echolocation frequency precluded species identification from video-recordings (G. Schreur and O. De Paz, *unpublished data*). Common movements between tunnels (maximum distance of 6.8 km) by radio-tracked individuals led us to consider the area’s bats as a single population.

Bats were caught in harp-traps as they entered the roost after foraging. Once identified, sexed, and aged, we placed them individually in cloth bags until sampling was carried out. Capture and handling protocols met the guidelines for treatment of animals in research and teaching [59], were approved by the Regional Council (license number: 0532041 PC 120), and met Spanish legal requirements. Bats were released at the roost after sampling. To minimise stress, retention time never exceeded 90 minutes. We discriminated between the two species based on the shape of the lancet and connecting process of the noseleaf. The lancet in *R*. *euryale* is essentially triangular, whereas in *R*. *mehelyi* it is notably concave laterally and very narrow distally. The connecting process is narrower and more pointed in *R*. *euryale*[25]. We restricted our analyses to adults, which were distinguished from juveniles by trans-illumination of the cartilaginous epiphyseal plates in the phalanges [60].

### Echolocation and wing parameters

Echolocation calls were recorded from bats held 30 cm from the microphone of a Pettersson D-980 ultrasonic detector (Pettersson Elektronik AB, Sweden). Signals were recorded into a laptop with ultrasound analysis software (BatSound 4.0; Pettersson Elektronik AB, Sweden). Because bats were held motionless, calls were not affected by Doppler shift; thus, the emitted frequency corresponded to the resting frequency (RF) [61]. Three-second samples were recorded at a rate of 448 kHz and time-expanded (10×). The resulting sequences of 30 seconds were then analysed using a sampling frequency of 44.1 kHz and a 1024-pt FFT [38]. We chose a random sample of ten echolocation signals from each individual, and measured the frequency of maximum energy from the power spectrum of a 10-ms portion of the constant frequency component of each call. Because horseshoe bats start emitting at a lower frequency before reaching the final RF level, we rejected starting signals for the analyses [41]. Starting signals were rejected manually in a spectrogram window after visual inspection. We calculated the mean frequency for the ten signals of each individual and used this mean for comparisons.

For each bat, we measured forearm length and body mass to the nearest 0.05 mm and 0.1 g, respectively. Wing morphology measurements included wingspan, wing area, aspect ratio, and wing loading. Wing area included the combined area of both wings, the entire tail membrane, and the body areas between the wings, excluding the head [17]. Aspect ratio is the square or the wingspan divided by the wing area and is related to energy efficiency. A higher aspect ratio corresponds to lower energy loss during flight. Wing loading is the weight of the bat divided by the area of its flight membrane and is correlated with flight speed. As wing loading increases, so too does the speed required to fly [17]. These variables were determined from wing tracings (to the nearest 1mm) on graph paper of the extended left wing. Tracings were scanned at 600 dpi and incorporated into a GIS (Arc View 3.2, ESRI, California, USA) for surface calculations.

To test for inter-specific differences in size and echolocation frequencies in sympatry we used multivariate ANOVA on arcosine-transformed measurements. Sex was introduced as a main factor in the analyses. We were unable to perform any statistical analysis with data on allopatry, since literature mostly provided mean values [17,24,25,38,40-42].

### Diet

We collected faecal samples, which were air-dried prior to analysis. Where possible, we randomly selected a minimum of five pellets from each individual; however, some bats did not excrete this many while handled (90 min). Pellets were soaked in water for 20 min and then teased apart with dissecting needles under a microscope. Based on arthropod fragments, we identified prey remains to the lowest taxonomic level possible using identification keys [62-64] and a reference collection. We estimated the percentage volume of each identifiable prey category visually to the nearest 5% for each pellet and then calculated a % for each individual [64]. We also estimated the relative sizes of moths consumed by *R*. *mehelyi* and *R*. *euryale* in sympatry. We extracted fragments of moth tarsus from the samples and measured the length of the first segment (LFS, from the joint with the second segment to the base of the claw) under a compound microscope to the nearest μm (magnification 32x). For specimens in our reference collection (*n* = 97), we measured moth body lengths (BL, excluding antennae and cerci) and LFS to obtain a regression equation (BL = 30.6LFS + 5.6; *r* = 0.95; F = 915.1; *d*.*f*. = 96; *p* < 0.001). Using this regression equation, we estimated the BL of moths consumed by bats based on LFS lengths in faecal samples.

To test for inter-specific differences in diet in sympatry and allopatry, we used a permutation test for two-way ANOVA between the proportions of prey consumed by both species. The significance of the *F*-statistic was obtained comparing the observed *F*-value to a reference distribution of *F*-values generated by 10000 permutations of the observed values between species [65]. Differences in estimated BL of consumed moths were tested using the Mann-Whitney *U* test.

### Radio-tracking

After clipping the fur from the mid sagittal dorsal surface, radio-transmitters (0.45 g; Pip II, Biotrack Ltd. Dorset, UK) were attached using surgical cement (Skinbond, Smith and Nephew, Largo, Florida, USA). We tagged 15 *R*. *mehelyi* and 16 *R*. *euryale*; from these we obtained radio-tracking data on 12 *R*. *mehelyi* and 13 *R*. *euryale* (Additional file 1: Appendix A). Radio-transmitter mass never exceeded 5% of the bat’s body mass [66]: percentage for the smallest *R*. *mehelyi* was 3.3% and for the smallest *R*. *euryale* was 4.0%. Before release at the roost, we checked whether bats were able to remove the transmitter and whether its position interfered with flight. The transmitter eventually fell off after 11-23 days (*personal observation*).

Bats were radio-tracked simultaneously by up to three mobile teams equipped with radio-receivers (1000-XRS, Wildlife Materials Inc., Carbondale, Illinois, USA, and FT290Rii, Andreas Wagener Telemetrienalegen, Köln, Germany) and hand-held three-element Yagi antennas. Mobile teams tracked bats from vehicles and on foot. Each night up to three observers equipped with radio-receivers located at different stationary vantage points guided the mobile teams to bats’ activity areas. The searching scope of the stationary observers encompassed almost all the study area, and they were able to detect the transmitter at a maximum distance of 10 km. Mobile and stationary trackers were coordinated using transceivers (VX-110, Yaesu Musen Co. Ltd., Japan) and cellular phones. Two or three bats were simultaneously tracked each night. Whenever possible, tracking was conducted continuously during the entire night. Bats were tracked by the “homing-in” technique, which involved following the bats to their activity areas as closely as possible and identifying their commuting routes and foraging areas *in situ*[67]. Bat locations were taken every 10 minutes to minimise spatiotemporal autocorrelation, because bats were able to commute to distant areas and had access to all habitat types with this time interval [67]. Subsequently, locations were transferred into a GIS database. Locations were recorded when bats were active and resting, but only active locations (hereafter referred to as foraging fixes) were used in our analyses. Individual foraging home ranges were determined by minimum convex polygons (MCPs) using individual foraging fixes. The colonial foraging range was determined as the MCP encompassing all foraging fixes of all individuals and was used to define the study area. This protocol is identical to that used by Salsamendi *et al*. [35] and Goiti *et al*. [34] to obtain radio-tracking locations in allopatric populations of *R*. *mehelyi* and *R*. *euryale*.

### Habitat use

For habitat use analyses, we included four environmental variables following Salsamendi *et al*. [35]: habitat type, canopy perimeter, canopy cover, and distance to water. Habitat types were mapped from local forest inventories and through photo-interpretation of ortho-photographs at a resolution of 4 pixels/m^2^. The resulting habitat-map was ground-truthed in the field. We identified ten habitat types: 1) pastures; 2) rice and corn fields; 3) scrublands, low vegetation dominated by Mediterranean maquis, interspersed with evergreen oaks; 4) dehesas, semi-natural savannah-like oak woodlands dominated by *Quercus rotundifolia* and *Q*. *suber*; 5) olive groves; 6) chestnut groves; 7) eucalypt plantations; 8) coniferous plantations; 9) riparian forests, woods along river banks dominated by *Populus* sp. and *Alnus glutinosa*—*Populus* sp. plantations, tree lines, and isolated trees near water courses were also included; and 10) broadleaved woodlands. Each foraging fix was assigned to a habitat type.

Canopy perimeter and canopy cover were quantified using GIS tools and used as an indication of habitat structure. We followed the next protocol: *i*) we transformed the original 4-pixels/m^2^-resolution ortho-photographs into 1-pixel/m^2^-resolution grey-scale geo-referenced images, in which each pixel had a value ranging from 0 to 255; *ii*) pixels representing trees (values from 1 to 100) were identified and selected at the attribute table, and a Boolean layer was generated from this selection (cut of levels were set after checking the range of values by visual inspection); *iii*) simultaneously, we modelled the study area as a randomly generated grid-layer (1 hectare per grid) covering the colonial MCP (41555 grids in total); and *iv*) we computed a geometric intersection between the tree (Boolean) layer and the grid layer to obtain a third layer, from which we obtained values of canopy perimeter and cover for each grid-cell. Thus, canopy cover was based on the sum of the areas of the pixels representing trees and indicated the percentage of tree cover for each 1-hectare grid-cell. Tree pixels connected to each other were aggregated into blocks and the perimeter of each block was measured. Canopy perimeter was based on the sum of the perimeters of the blocks and indicated the amount of edge habitat for each 1-hectare grid-cell. Each foraging fix in this study (sympatric condition) as well as those obtained by Salsamendi *et al*. [35] for *R*. *mehelyi* and by Goiti *et al*. [34] for *R*. *euryale* (both in allopatry), were assigned to a single grid cell canopy cover and perimeter values. Differences in canopy cover and canopy perimeter values between *R*. *mehelyi* and *R*. *euryale* in sympatric and allopatric conditions were assessed on square root-transformed values using one-way ANOVAs.

Onto a separate layer, we identified natural and artificial water bodies (rivers, wetlands, canals, and ponds for irrigation and livestock) within the colonial MCP from photo-interpretation of ortho-photographs and observations in the field. Minimum width for rivers was set to 1 m and minimum area for water bodies to 4 m^2^ to be included in the analyses. The corresponding distance from foraging fixes to the nearest water source was included in the analyses and was used as an indication of water availability. We used one-way ANOVA to determine if there was an association between canopy perimeter, canopy cover and habitat type. Repeated-measures ANOVA was used to test for inter-specific differences in mean canopy perimeter, canopy cover, and distance to water.

We used Classification and Regression Tree (CART) analysis to model differences in foraging habitat preferences between *R*. *mehelyi* and *R*. *euryale* in sympatry. CART is a statistical method to construct binary trees promoted as a strong tool for predictive modelling [68]. Splits are based on cut-off levels of the predictors, which produce maximum separation between two data subgroups and minimum variability within them with respect to the outcome. These splits are determined from a search of all possible cut-off points, and only significant splits (*p*< 0.05) were used in the final model [69]. Foraging fixes of both species were randomly divided, with 60% used as training data to select the best model and 40% retained for model validation. The response variable was *R*. *mehelyi*/*R*. *euryale* foraging fix, whereas the habitat type, canopy perimeter, canopy cover, and distance to water were explanatory variables. To avoid over-fitting the data, we used a pruning methodology that determined a nested sequence of sub-trees by recursively removing the least-important splits from the full tree. We selected the optimal tree based on cross-validation [70]. We evaluated the predictive ability of the CART model by calculating the *c* statistic and 95% confidence interval from a logistic regression model in the validating model. By cross-tabulation, we calculated the percentage of foraging fixes correctly predicted for each species in the validation sample.

### Niche breadth and overlap

We computed habitat and trophic niche breadth by means of Levins’ index B [71]: B_i_ = 1/(Σp_ir_^2^), where B_i_ is the Levins’ index of individual *i*. For habitat niche breadth, p_ir_ is the proportion of *r* habitat type used by individual *i*, whereas for trophic niche breadth, p_ir_ is the proportion of *r* prey category consumed by individual *i*. Levins’ index ranges from 1 (one habitat/prey category used) to *n* (total number of habitat/prey categories). We also computed intra-specific and inter-specific habitat and dietary niche overlap using the Freeman-Tukey index [72]: FT_ij_ = Σ (p_ir_ · p_jr_)^1/2^, where FT_ij_ is the Freeman-Tukey measure of niche overlap between individuals *i* and *j*. For habitat niche overlap, p_ir_ and p_jr_ are the proportions of *r* habitat types used by individuals *i* and *j*, whereas for trophic niche overlap, p_ir_ and p_jr_ are the proportions of *r* prey category consumed by individuals *i* and *j*. This measure of overlap ranges from 0 (no habitat/prey used in common) to 1 (complete overlap in habitat/prey used). For intra-specific niche breadth and overlap, we calculated the measure for every possible pair of individuals within one species. For inter-specific niche overlap, we calculated the measure for every possible pair of individuals of both species. Differences in both niche breadths and overlaps relied on randomisation procedures to avoid the statistical pseudo-replication inherent in pairwise comparisons [73]. To test for differences between niche breadths and overlap means, we carried out two-sample randomisation tests. The significance of the *t*-statistic was obtained by comparing the observed *t*-value to a reference distribution of *t*-values generated by 10000 permutations of the observed values between the two species. We computed niche and breadth overlap measures for sympatric data on habitat use and diet.

Statistical analyses were performed using SAS v.9.2 (SAS Institute Inc., Cary, USA), except CART analysis was conducted using R software v.2.14 (R Foundation for Statistical Computing, Vienna, Austria). For all tests we assumed a significant result when alpha < 0.05.

## Abbreviations

RF: Resting frequency: echolocation call frequency of maximum energy emitted by horseshoe bats when resting; BL: Body length measure of a moth’s body length excluding antennae and cerci; B: Levins’ index (B) computed to calculate habitat and trophic niche breadth; FT: Freeman-tukey index (FT) computed to calculate habitat and trophic overlap; GIS: Geographic information system; MCP: Minimum convex polygon; CART analysis: Classification and regression tree analysis.

## Competing interests

The authors declare that they have no competing interests.

## Authors’ contributions

ES, IG and JA conceived of, designed, and coordinated the study. IA participated in the design of the study and performed the statistical analyses. UG participated in the design of the study and in fieldwork. All authors read and approved the final manuscript.

## Supplementary Material

Additional file 1 **Appendix A.** Radio-tracking details of *R*. *mehelyi* and *R*. *euryale* individuals followed in sympatry; Sex, reproductive status (pregnant females, lactating females), tracking effort (tracking period and active foraging fixes [AFF]), individual foraging home ranges (MCP 100%), and travelled distances (maximum and mean) of radio-tracked *R*. *mehelyi* and *R*. *euryale* individuals in sympatric conditions. Click here for file
