# Supplementary material for: What mechanism of niche segregation allows the coexistence of sympatric sibling rhinolophid bats?
Source: Front Zool. 2012 Nov 13;9:30. doi: 10.1186/1742-9994-9-30 (PMC3542077; doi:10.1186/1742-9994-9-30)
Supplement: Additional file 1 — Appendix A. Radio-tracking details of R. mehelyi and R. euryale individuals followed in sympatry; Sex, reproductive status (pregnant females, lactating females), tracking effort (tracking period and active foraging fixes [AFF]), individual foraging home ranges (MCP 100%), and travelled distances (maximum and mean) of radio-tracked R. mehelyi and R. euryale individuals in sympatric conditions. [file 1742-9994-9-30-S1.docx]

**Additional file 1: Appendix 1**

Appendix 1; **Radio-tracking details of *R. mehelyi* and *R. euryale* individuals followed in sympatry**; Sex, reproductive status (pregnant females, lactating females), tracking effort (tracking period and active foraging fixes [AFF]), individual foraging home ranges (MCP 100%), and travelled distances (maximum and mean) of radio-tracked *R. mehelyi* and *R. euryale* individuals in sympatric conditions.

|  |  |  | Tracking effort | |  | Distance (km) | |
| --- | --- | --- | --- | --- | --- | --- | --- |
| Code | Sex | Repr. status | Tracking period | AFF | MCP 100% (ha) | Max. | Mean |
| *R. mehelyi* | | | | | | | |
| 149 | ♂ |  | 13-16 June | 15 | 345 | 3.9 | 1.6 |
| 303 | ♀ | lactating | 30 June–4 July | 13 | 449 | 29.1 | 27.5 |
| 363 | ♀ | lactating | 29 June | 14 | 68 | 23.1 | 23.1 |
| 403 | ♂ |  | 11-13 July | 23 | 19 | 17.9 | 17.7 |
| 453 | ♂ |  | 11-13 July | 23 | 1 | 10.0 | 9.9 |
| 483 | ♂ |  | 28 June–3 July | 21 | 174 | 21.1 | 20.6 |
| 543 | ♀ | lactating | 30 June | 12 | 40 | 20.9 | 20.3 |
| 555 | ♂ |  | 12-13 July | 17 | 121 | 22.4 | 19.1 |
| 573 | ♀ | lactating | 2-3 July | 18 | 0.2 | 25.9 | 25.8 |
| 679 | ♂ |  | 11-13 July | 23 | 358 | 22.7 | 21.3 |
| 844 | ♀ | lactating | 26 June | 17 | 147 | 21.9 | 20.6 |
| 871 | ♂ |  | 12-13 July | 18 | 96 | 22.7 | 22.3 |
| Mean (SD) |  |  |  | 17.8 (3.9) | 242 (341) | 20.1 (6.8) | 19.2 (7.9) |
| *R. euryale* | | | | | | | |
| 045 | ♂ |  | 16 June | 12 | 7 | 7.6 | 7.5 |
| 065 | ♂ |  | 17-19 June | 44 | 22 | 4.9 | 4.6 |
| 078 | ♀ | pregnant | 18 June | 26 | 20 | 6.8 | 6.6 |
| 089 | ♀ | pregnant | 19-20 June | 35 | 124 | 4.3 | 4.1 |
| 116 | ♂ |  | 16-18 June | 23 | 55 | 3.6 | 2.9 |
| 182 | ♂ |  | 14-20 June | 44 | 1124 | 7.2 | 5.2 |
| 208 | ♂ |  | 13-17 June | 20 | 290 | 10.1 | 8.1 |
| 220 | ♀ | lactating | 3 July | 2 | – | 1.9 | 1.9 |
| 231 | ♂ |  | 13-15 June | 13 | 33 | 1.3 | 0.9 |
| 266 | ♂ |  | 13-18 June | 33 | 35 | 5.5 | 5.0 |
| 377 | ♀ | lactating | 1 July | 25 | 4 | 5.9 | 5.8 |
| 808 | ♀ | lactating | 26-27June | 25 | 96 | 10.1 | 9.5 |
| 856 | ♀ | lactating | 1 July | 23 | 30 | 4.4 | 3.6 |
| Mean (SD) |  |  |  | 25 (12.1) | 153 (316) | 5.6 (2.7) | 4.8 (2.4) |
